# Reconstruction of the Knee Extensor Mechanism Using a Semitendinosus Tendon Graft and a Free Vascularized Latissimus Dorsi Musculocutaneous Flap for Complete Extensor Mechanism Loss With an Extensive Soft Tissue Defect: A Case Report

**DOI:** 10.7759/cureus.102337

**Published:** 2026-01-26

**Authors:** Kenichi Otoshi, Soichi Ejiri, Hironori Numazaki

**Affiliations:** 1 Sports Medicine, Fukushima Medical University, Fukushima, JPN; 2 Orthopaedic Surgery, Fukushima Medical University School of Medicine, Fukushima, JPN

**Keywords:** complete patellar defect, knee extensor mechanism, latissims dorsi musculocutaneus flap, reconstruction, semitendinosus tendon

## Abstract

A 58-year-old male sustained an injury that resulted in complete loss of the knee extensor mechanism, with an extensive soft tissue defect on the anterior aspect of the knee. Reconstruction of the knee extensor mechanism using an autologous semitendinosus tendon graft and coverage of the soft tissue defect using a free vascularized latissimus dorsi musculocutaneous flap were performed. The patient returned to his previous work six months after surgery without difficulty, except for a slight feeling of exhaustion when going up and down stairs. Recovery of hamstring muscle strength was sufficient, whereas quadriceps muscle strength remained less than 30% compared with the contralateral side even two years after surgery.

## Introduction

The extensor mechanism of the knee consists of the quadriceps muscles, quadriceps tendon, patella, and patellar tendon. Many reports have described the etiology, management, and surgical techniques used to address disruption of the extensor mechanism [1-3].

High-velocity open knee injuries sometimes result in varying degrees of knee extensor mechanism disruption (e.g., inability to extend the knee), with severe soft tissue damage around the knee joint. A gastrocnemius musculocutaneous flap has been used to cover the soft tissue defect and substitute for the knee extensor mechanism in such cases [4,5], but direct reconstruction of the knee extensor mechanism and coverage of the soft tissue defect with a free vascularized musculocutaneous flap has not been previously reported. In the present case, extensor mechanism reconstruction was performed using autologous semitendinosus tendons for traumatic, complete extensor mechanism loss, with simultaneous coverage of the co-existing extensive soft tissue defect on the anterior aspect of the knee using a free vascularized latissimus dorsi musculocutaneous flap.

This article was previously presented as a meeting abstract at the 15th ESSKA Congress on May 2-5, 2012.

## Case presentation

**Figure 1:**
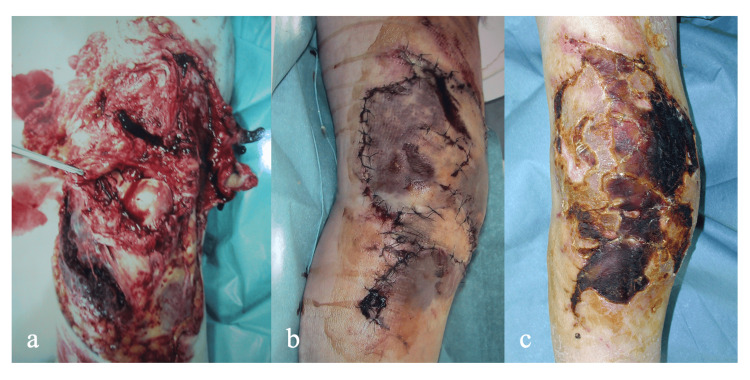
Outward appearance of the right knee. A: At the time of injury B: Immediately after the first surgery C: Just before the second surgery A large soft tissue defect is present on the anterior aspect of the knee. The patella and patellar tendon are completely destroyed, and the distal end of the quadriceps tendon is severely damaged (A). Debridement was performed immediately under spinal anesthesia, and primary wound closure was performed using residual skin (B). However, the wound edge gradually became necrotic (C).

**Figure 2:**
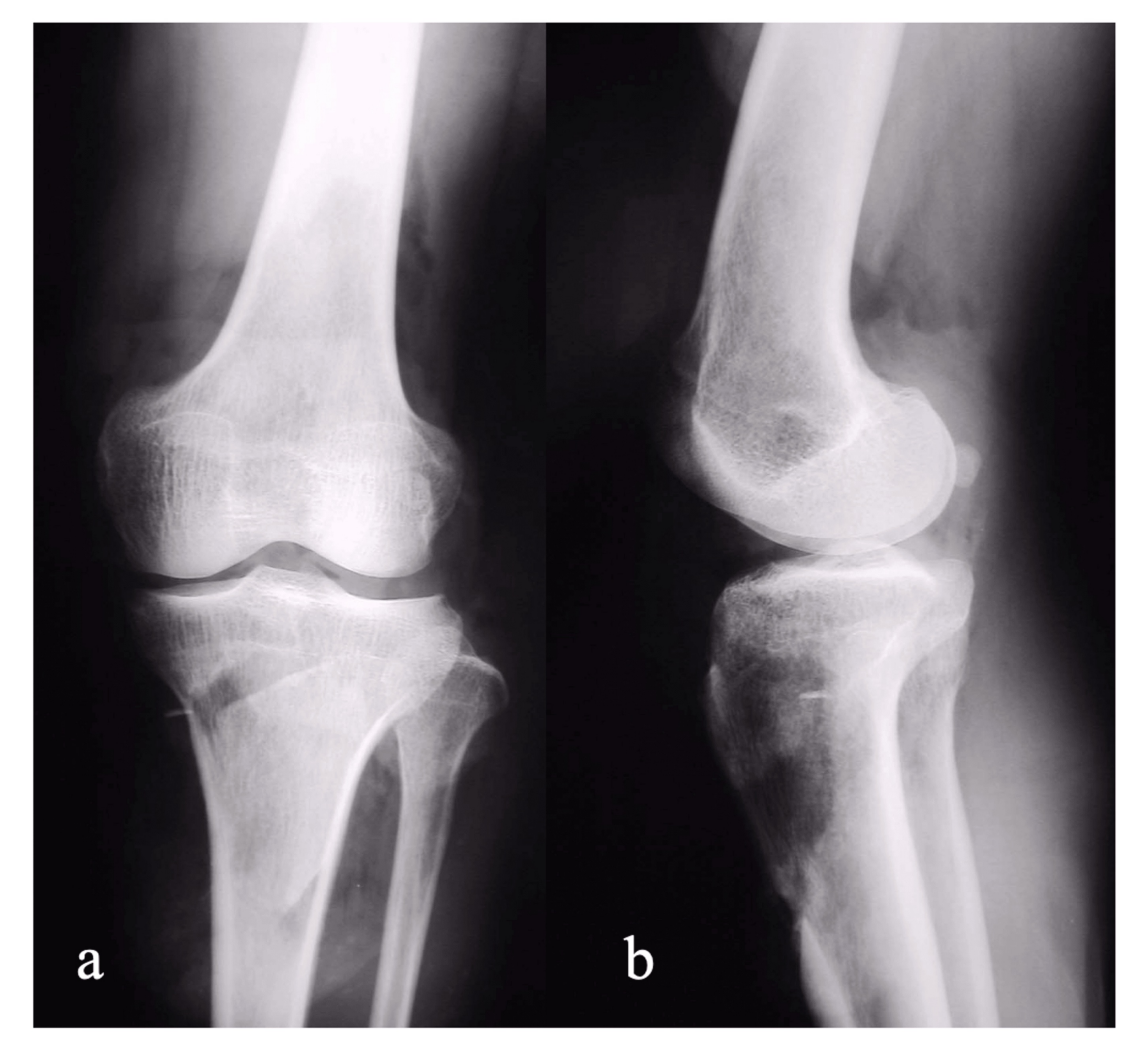
Plain X-ray of the right knee. A: AP view B: Lateral view A plain X-ray of the knee shows a patellar deficit, with no remarkable bony damage. AP: Antero-posterior.

A 58-year-old male injured his right leg while operating a snowplow and was immediately taken to the emergency center of our hospital. He complained of severe right knee pain, and he could not straighten his knee actively. There was a large soft tissue defect on the anterior aspect of the knee. The patella and patellar tendon were completely destroyed, and the quadriceps tendon was severely damaged (Figure 1A). A plain X-ray of the knee showed a patellar deficit, while there was no bony damage (Figure 2A-2B). Debridement was performed immediately under spinal anesthesia, and primary wound closure was done using residual damaged skin (Figure 1B), but the skin gradually became necrotic (Figure 1C).

**Figure 3:**
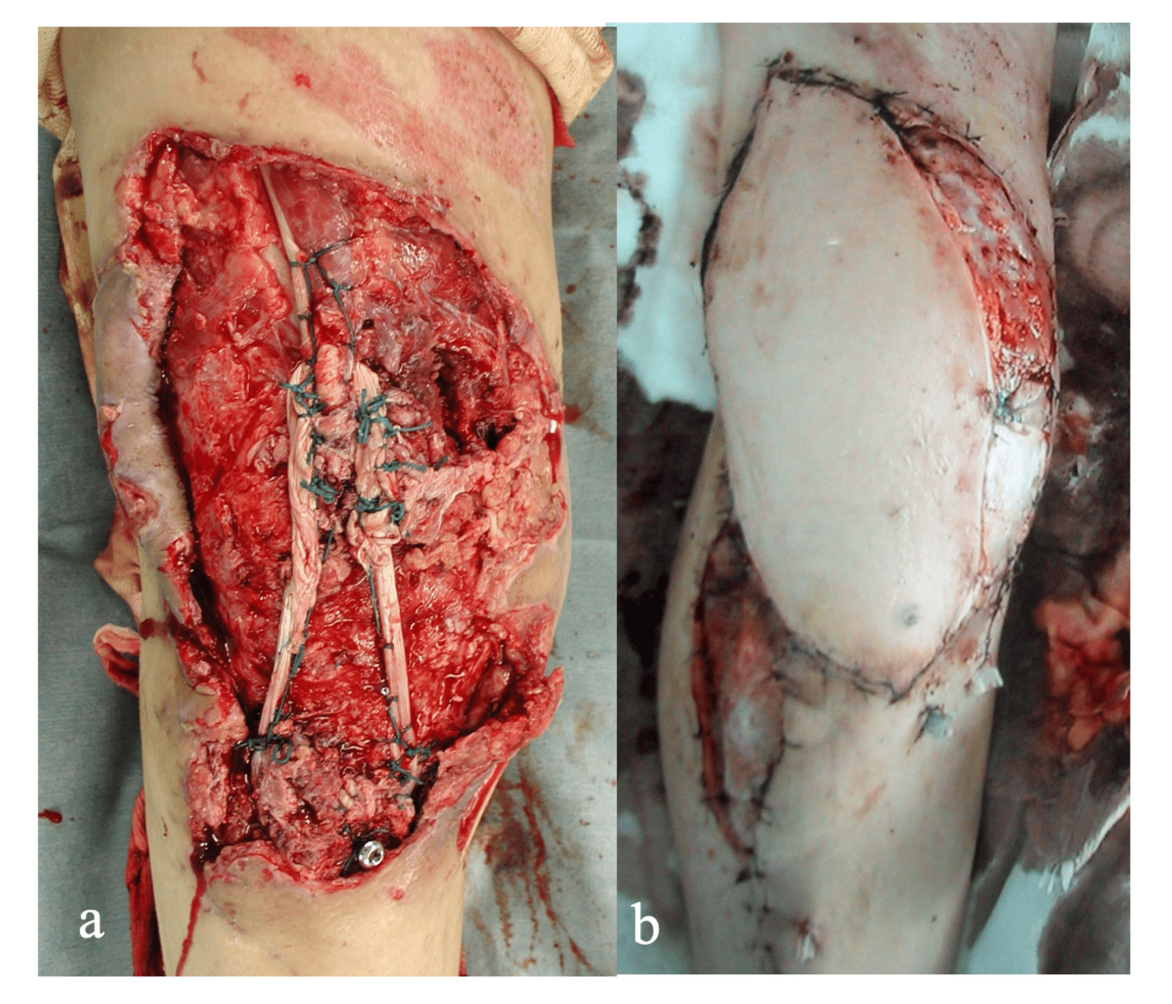
Reconstruction of the extensor mechanism and soft tissues. A: Extensor mechanism reconstruction B: Coverage of the soft tissue defect Bilateral autologous semitendinosus tendons were used for reconstruction of the extensor mechanism (A), and a free vascularized latissimus dorsi musculocutaneous flap with a free skin graft was used to cover the large soft tissue defect (B).

The active range of motion was limited from 20 to 70 degrees. This condition was diagnosed as dysfunction of the knee extensor mechanism, and simultaneous reconstruction of the knee extensor mechanism and soft tissue coverage of the anterior aspect of the knee were planned. The operation was performed one month after the initial injury. The size of the skin necrosis was approximately 250 mm long and 100 mm wide. Necrotic skin and soft tissues were removed, and the distal end of the quadriceps tendon was identified and released. The semitendinosus tendons were used for the graft. Bilateral semitendinosus tendons were stripped using a tendon stripper. The contralateral semitendinosus tendon was isolated, whereas the tibial insertion of the ipsilateral semitendinosus tendon was preserved. A horizontal bone tunnel was made in the right tibia just distal to the tibial tuberosity from lateral to medial, taking care to preserve the insertion of the semitendinosus tendon. The distal end of the contralateral semitendinosus tendon was passed through the bone tunnel from lateral to medial and fixed to the medial side of the right tibia with a post screw. The free ends of both semitendinosus tendons were pulled proximally and interlaced with the distal end of the quadriceps tendon while applying traction to the quadriceps muscles, adjusting the tension and knee range of motion (Figure 3A). A free vascularized musculocutaneous flap was used to cover the soft tissue defect on the anterior aspect of the knee. A free vascularized musculocutaneous flap (220 mm long, 150 mm wide, 18 mm thick) was harvested from the ipsilateral latissimus dorsi muscle. After performing anastomoses between the thoracodorsal vessels and tibial anterior vessels under microscopy, the flap was transplanted to the soft tissue defect. Free skin grafts were added to cover the exposed latissimus dorsi muscle and tibialis anterior muscle (Figure 3B).

**Figure 4:**
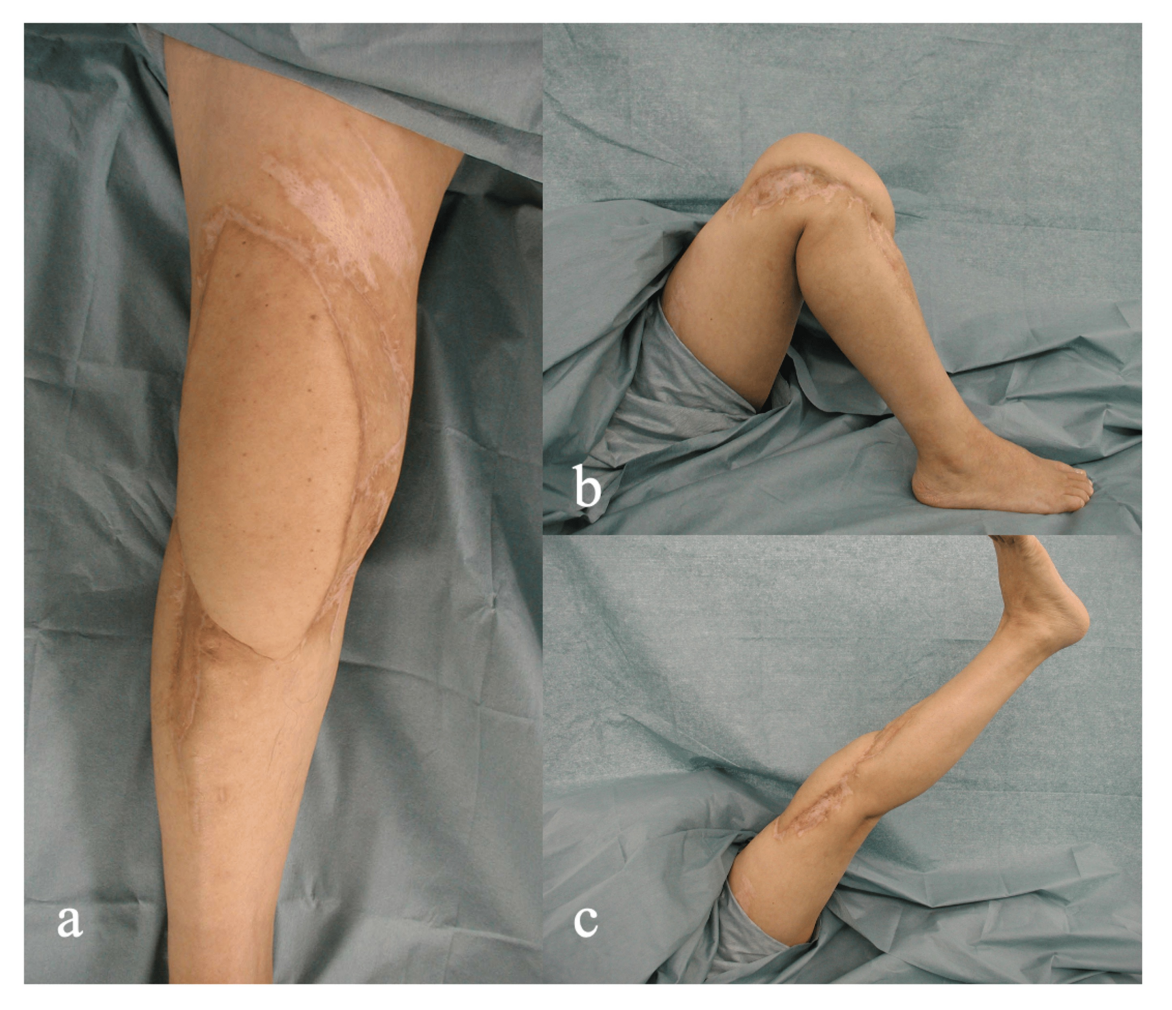
Outward appearance of the right knee 2 years after surgery. A: Anterior aspect B: Lateral aspect with the knee in full flexion C: Lateral aspect with the knee in full extension Engraftment of the flap was confirmed 2 years after surgery (A). The passive range of motion is 0-120 degrees, and the patient can extend his knee without extension lag (B, C).

The knee was fixed with a splint in extension. Anticoagulant therapy was continued for one week after surgery. After stable circulation of the flap was confirmed, isometric quadriceps and hamstring muscle exercises were started. Partial weight-bearing walking exercise with a knee brace was allowed two weeks after surgery, and full weight-bearing and passive range of motion exercises were started four weeks after surgery. The patient could walk without a brace or crutch 6 months after surgery. The passive range of motion was 0-120 degrees, and he could extend his knee without extension lag (Figure 4A-4C).

**Figure 5:**
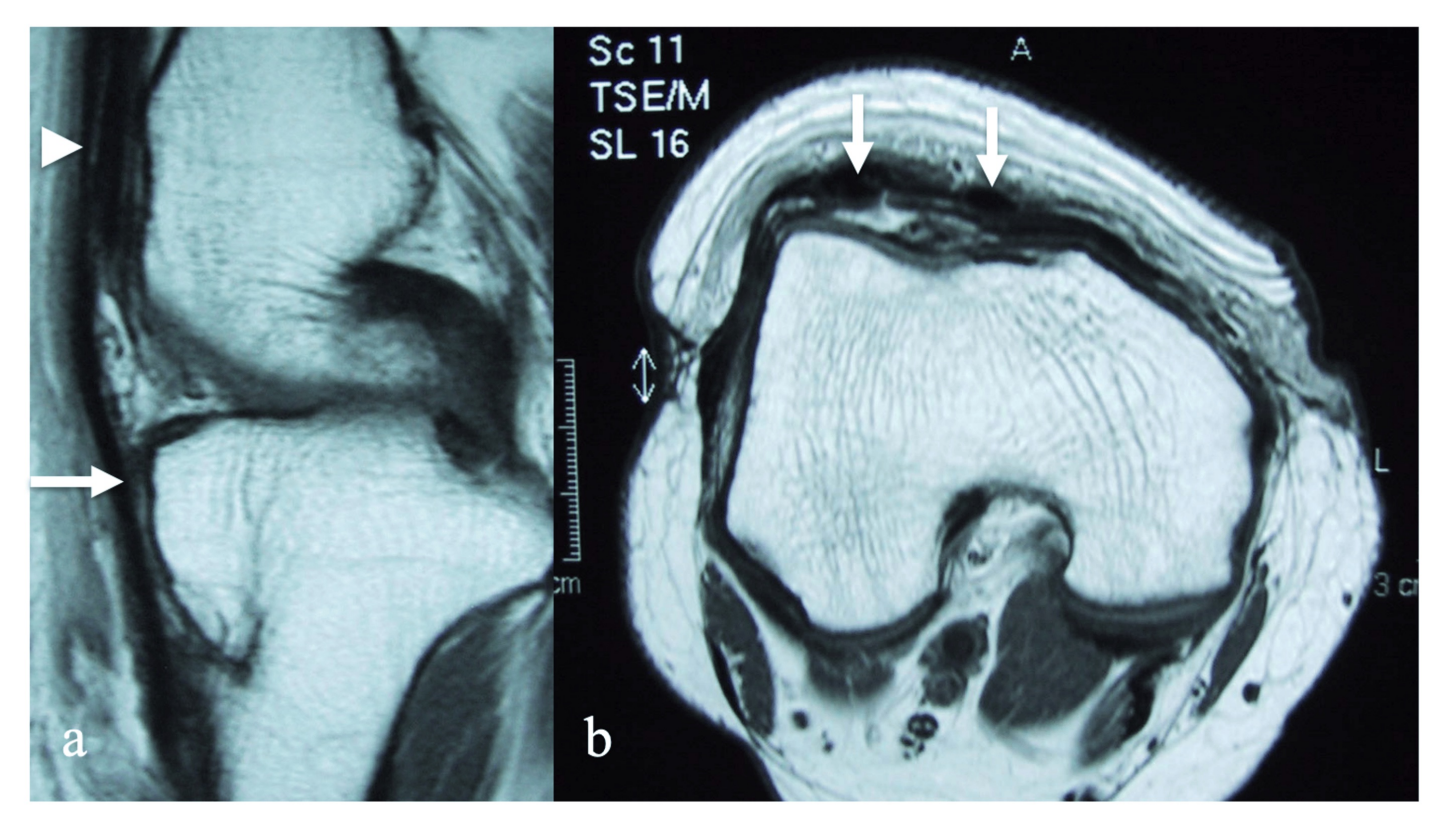
MRI of the right knee 1 year after surgery. A: Sagittal view B: Axial view Two low-signal-intensity bundles (arrows) are identified between the tibial tuberosity and the distal end of the quadriceps tendon (arrowhead) within the femoral trochlea.

The patient could return to his previous work without any difficulty, with the exception of a slight feeling of exhaustion while going up and down stairs. Isokinetic hamstring and quadriceps muscle strength was measured using an isokinetic dynamometer (KinCom, Chattanooga, TN, USA). Hamstring muscle strength on the affected side was 70.7%, 72%, and 81% compared with the unaffected side at 6 months, 1 year, and 2 years after surgery, respectively, and quadriceps muscle strength was 23.1%, 28.3%, and 22.2% on the affected side, respectively (Table 1). Hamstring strength improved gradually, but there was little improvement in quadriceps muscle strength even 2 years after surgery. MRI showed two low-signal-intensity bundles, which were considered to be the grafted semitendinosus tendons, between the tibial tuberosity and the distal end of the quadriceps tendon within the femoral trochlea (Figure 5A-5B).

**Table 1:** Hamstring and quadriceps muscle strength. Hamstring muscle strength on the affected side was 70.7%, 72%, and 81% compared with the unaffected side at 6 months, 1 year, and 2 years; however, quadriceps muscle strength on the affected side was 23.1%, 28.3%, and 22.2%, respectively. Hamstring strength improved gradually, but there was little improvement in quadriceps muscle strength even 2 years after surgery. Isokinetic dynamometer (KinCom, Chattanooga, TN, USA); 90 degrees/second.

	Hamstring muscle strength			Quadriceps muscle strength		
	Affected side (N)	Unaffected side (N)	A/U ratio (%)	Affected side (N)	Unaffected side (N)	A/U ratio (%)
6 months	133	158	70.7	78	338	23.1
1 year	157	218	72	107	379	28.3
2 years	158	195	81	100	450	22.2

## Discussion

Several reports have described successful results using a gastrocnemius muscle flap to reconstruct the knee extensor mechanism and cover the soft tissue defect on the anterior aspect of the knee [4-6]. In a small case series of gastrocnemius flaps for traumatic knee extensor disruption with a soft tissue defect, the size of the defect after debridement ranged from 8×5 cm to 15×15 cm, and both gastrocnemii were used if the soft tissue defect was over 10×10 cm [4]. Given the previous reports, we decided to use a free vascularized latissimus dorsi musculocutaneous flap because the defect size was considered too large to cover with a gastrocnemius muscle flap, even if both gastrocnemii were used. With this flap, it was easy to adjust the size according to the defect size, and it had sufficient thickness to protect the knee structures.

Whereas a free vascularized re-innervated musculocutaneous flap has been used for reconstruction of the knee extensor mechanism [7,8], we considered that it would not substitute sufficiently for the knee extensor mechanism, and that there was a need to reconstruct the knee extensor mechanism independently. It has been reported that autologous tendons, allografts, or artificial tendons have been used for augmentation or reconstruction of the extensor mechanism [1-3,9,10]. However, it is considered difficult to reconstruct the knee extensor mechanism in the presence of a patellar defect. Only two reports have described reconstruction of extensor mechanism disruption for combined patella, patellar tendon, and quadriceps tendon defects [9,10]. One report described reconstruction using a hamstring allograft for quadriceps disruption after patellectomy [10], and the other described reconstruction using a tibial tuberosity-patellar tendon-patella-quadriceps tendon allograft for dysfunction of the knee extensor mechanism after total knee arthroplasty in a patient who had undergone patellectomy [9]. Since allograft use is limited due to ethical considerations in our country and there is a risk of infection, we decided to use a semitendinosus tendon autograft. The semitendinosus tendon graft had sufficient length, and it was easy to adjust the gap between the tibial tuberosity and the quadriceps tendon. It has been reported that the mean ultimate tensile strength of a double-bundle semitendinosus tendon is approximately 2330 N, and that the maximum tensile load of the patellar tendon when the quadriceps isotonically contracts maximally is 2800 N [11,12]. According to these reports, two strands of semitendinosus tendon may have almost enough tensile strength to substitute for the extensor mechanism clinically.

It has been reported that the main biomechanical function of the patella is to increase the moment arm of the quadriceps mechanism [13]. In the present case, ipsilateral quadriceps muscle strength was less than 30% compared with the contralateral side even 2 years after surgery. It has been reported that knee extension ability decreases by approximately 50% compared with the normal side after patellectomy [14], and that patients with a loss of peak flexion torque of more than 30% show an unsatisfactory clinical result [15]. In the present case, it would appear that sufficient recovery of knee flexion strength might have contributed to the good clinical outcome, whereas recovery of quadriceps muscle strength was insufficient.

## Conclusions

Combined reconstruction of the knee extensor mechanism using an autologous hamstring tendon, with coverage of the soft tissue defect using a free vascularized musculocutaneous flap, appears to be a treatment option for extensive loss of the knee extensor mechanism with a soft tissue defect on the anterior aspect of the knee. Hamstring tendons have sufficient strength, and their length is easy to adjust according to the gap, and a free vascularized latissimus dorsi musculocutaneous flap has sufficient size and thickness regardless of the defect size.
